# New Insights From Single-Cell Sequencing Data: Synovial Fibroblasts and Synovial Macrophages in Rheumatoid Arthritis

**DOI:** 10.3389/fimmu.2021.709178

**Published:** 2021-07-19

**Authors:** Liyun Cheng, Yanyan Wang, Ruihe Wu, Tingting Ding, Hongwei Xue, Chong Gao, Xiaofeng Li, Caihong Wang

**Affiliations:** ^1^ Department of Rheumatology, the Second Hospital of Shanxi Medical University, Taiyuan, China; ^2^ Pathology, Joint Program in Transfusion Medicine, Brigham and Women’s Hospital/Children’s Hospital, Harvard Medical School, Boston, MA, United States

**Keywords:** single-cell RNA sequencing, rheumatoid arthritis, synovial fibroblast, synovial macrophage, biomarkers

## Abstract

Single-cell RNA sequencing (scRNA-seq) technology can analyze the transcriptome expression level of cells with high-throughput from the single cell level, fully show the heterogeneity of cells, and provide a new way for the study of multicellular biological heterogeneity. Synovitis is the pathological basis of rheumatoid arthritis (RA). Synovial fibroblasts (SFs) and synovial macrophages are the core target cells of RA, which results in the destruction of articular cartilage, as well as bone. Recent scRNA-seq technology has made breakthroughs in the differentiation and development of two types of synovial cells, identification of subsets, functional analysis, and new therapeutic targets, which will bring remarkable changes in RA treatment.

## Introduction

Rheumatoid arthritis (RA) is a progressive aggressive immune disorder which can lead to increased mobility and disability, the main features of this disease are persistent synovitis, pannus formation, joint destruction, and adjacent bone erosions (1–3). At present, it is considered that environmental and genetic factors are related to the pathogenesis of RA, its etiology and pathogenesis have not been fully elucidated and remain to be clarified (4). The traditional therapeutic approaches of RA are suppressing the excessive immunological response and inflammatory reaction, which can only help to relieve RA symptoms and delay disease progression rather than cure (5). In addition, these strategies lead to several systemic side effects (6). Therefore, to explore the effective molecular targets for the treatment of RA is a focus of research (7).

ScRNA-seq is a technology for high-throughput sequencing and analysis of genome and transcriptome at single-cell level. It finds rare new cell subpopulations and shows the changes in each cell. It is a new technology to study the genetic heterogeneity of multicellular organisms which plays increasingly remarkable role in cancer research, developmental biology and neuroscience (8–10). With regards to rapid development of ScRNA-seq, the development lineage of immune cells is revealed in the field of autoimmune diseases, and the gene modules and regulatory procedures that determine the immune response are identified (11, 12). The latest research has reported the heterogeneity of synovial fibroblasts and macrophages in RA and the differences of their origins and biological characteristics. The purpose of this paper is to further clarify the pathogenesis of RA from the single cell biological level and explore new therapeutic breakthroughs.

Whether the tumor like abnormal proliferation and erosiveness of RA synovial fibroblasts are related to their heterogeneity has always been the focus of scholars’ research. it is well known that the different functions occur of fibroblasts in different anatomical site (13). However, there is no unified and comprehensive classification of synovial fibroblasts, and the specific markers of different subpopulations, and their specific roles in the pathological process of RA have not been fully elucidated. Single cell sequencing can maximize the genetic information of a single cell and discover the heterogeneity between cells. According to different algorithms, cells with different commonness are divided into different subpopulations, and compared with the known library to identify specific cell markers of different communities, and further discover and identify new cell surface markers. Compared with synovial fibroblasts, the action of synovial macrophages in the pathophysiology and pathological processes of RA is poorly understood. Because the number of synovial macrophages is limited and difficult to obtain, the origination and exact function of synovial macrophages in inflammatory diseases are not fully known (14).With the development of ScRNA-seq in recent years, the research of synovial fibroblasts has stepped to a new level. ScRNA-seq can study the development spectrum of synovial fibroblasts and macrophages on the basis of single cell, and dynamically analyze cell heterogeneity. It plays an imperative role in finding new targets for RA treatment, and has become a significant research approach. The core steps of ScRNA-seq technology and its application in the research of synovial fibroblasts and macrophages in RA will be introduced in this paper.

## Single Cell Sequencing Technology

The development and application of next-generation sequencing technology have brought great changes to the development of biological research. However, the results obtained by traditional population sequencing are the average value of the whole sample, or reflect the data of the dominant number of cells. In 2009, Tang et al. (15) achieved the first single cell transcriptome sequencing by improving the previously used single cell transcriptome amplification method for microarray analysis. With the development of sequencing, cell separation and genome-wide amplification, single cell sequencing technology has been improved and is becoming the focus of life science research.

Single-cell sequencing (SCS) has mushroomed as a powerful novel set of technologies in NGS, consisting of single-cell DNA sequencing, scRNA-seq, along with single-cell epigenomic sequencing (16). Among them, scRNA-seq, which is the most widely used, can reveal the subtle changes of transcriptome of each cell, clarify the heterogeneity of mRNA expression among individual cells, and obtain more sample information by highly efficient amplification and high-throughput sequencing. At the same time, scRNA-seq technology can solve the problem of low sample acquisition. Hundreds to thousands of cells can meet the needs of sequencing. The sample size is flexible. It plays an indispensable role in revealing the source and function of cells, finding new functional cell subsets and therapeutic targets.

**Figure 1:**
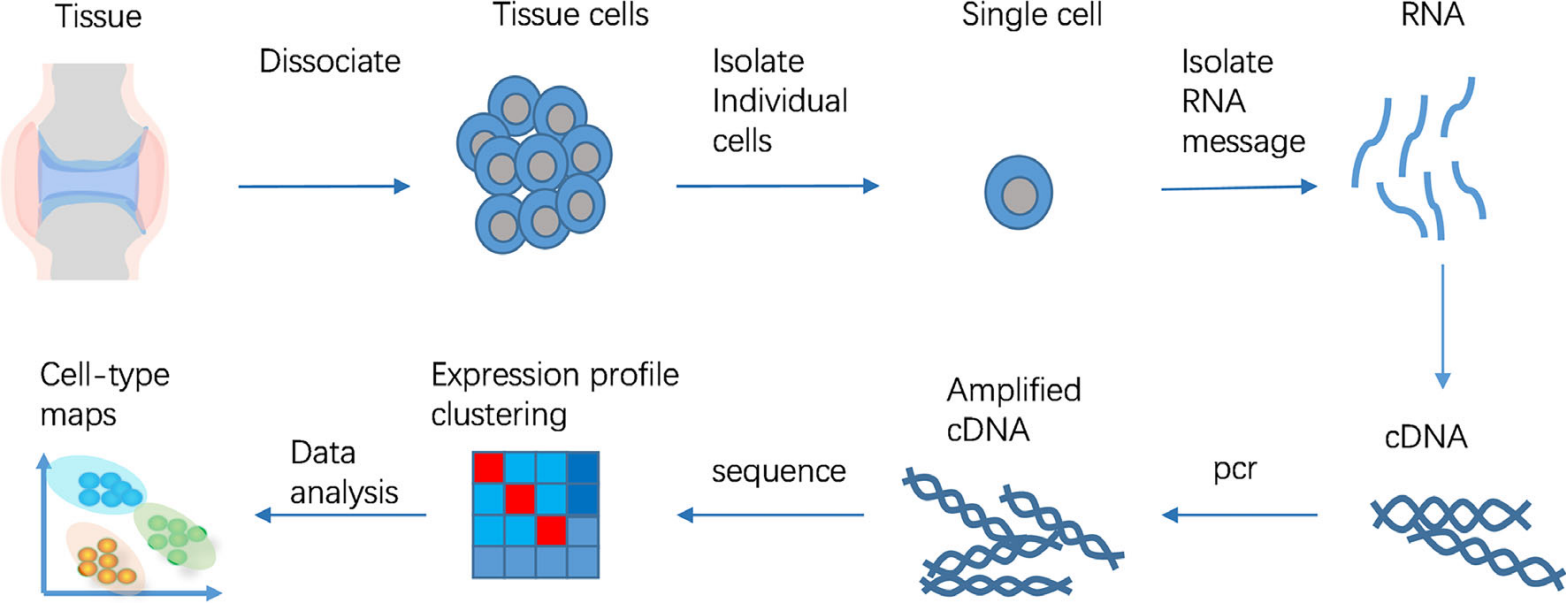
ScRNA-seq workflow. The ScRNA-seq process includes isolation and cleavage of single cell or single cell nucleus, reverse transcription, cDNA amplification, construction of sequencing library.

ScRNA-seq is used to amplify and sequence mRNA at a single cell level through high-throughput detection. Generally, it includes the following steps: (1) isolation and cleavage of single cell or single cell nucleus; (2) reverse transcription; (3) cDNA amplification; (4) construction of sequencing library (17)(**Figure 1**). The separation and capture of single cell and reverse transcription and amplification of cDNA with minimally mRNA are the two key points in the whole technical process. The development of ScRNA-seq technology largely depends on the continuous optimization of the solutions to these two problems. The frequently utilized single cell separation approaches include continuous dilution, magnetic activated cell sorting, micromanipulation, fluorescence activated cell sorting, microfluidic platform, and laser capture microdissection (18). Because of the small sample size of ScRNA-seq technology, the optimization of experimental process and the accuracy of steps are of great significance. Different cell capture methods, cDNA amplification and library construction methods are being reported and used. New ScRNA-seq platform is also constantly improving. ScRNA-seq technology is developing towards the direction of gradually reducing the cost and increasing the throughput.

## ScRNA-seq of Synovial Fibroblasts

### Function of Synovial Fibroblasts

In health the joint synovium is a delicate and thin structure, which encapsulates articular joints and functions as a homeostatic balance of the synovial fluid for efficient and smooth movement. The structure of synovium can be divided into two layers: the sublining, as well as lining layer of which in a healthy joint constitutes of one to two cell layers thick. There is no obvious boundary between the two layers. The synovial intimal lining directly interfaces with the synovial fluid and is composed of spindle-shaped fibroblasts aligned in a cohesive layer. Macrophages are distributed in the fibroblast layer. This layer enhances the barrier role, as well as secretes hyaluronic acid and lubricin. Less densely packed fibroblasts along with macrophages in a loose tissue matrix coupled with a network of blood vessels make up the sublining layer (19).

**Figure 2:**
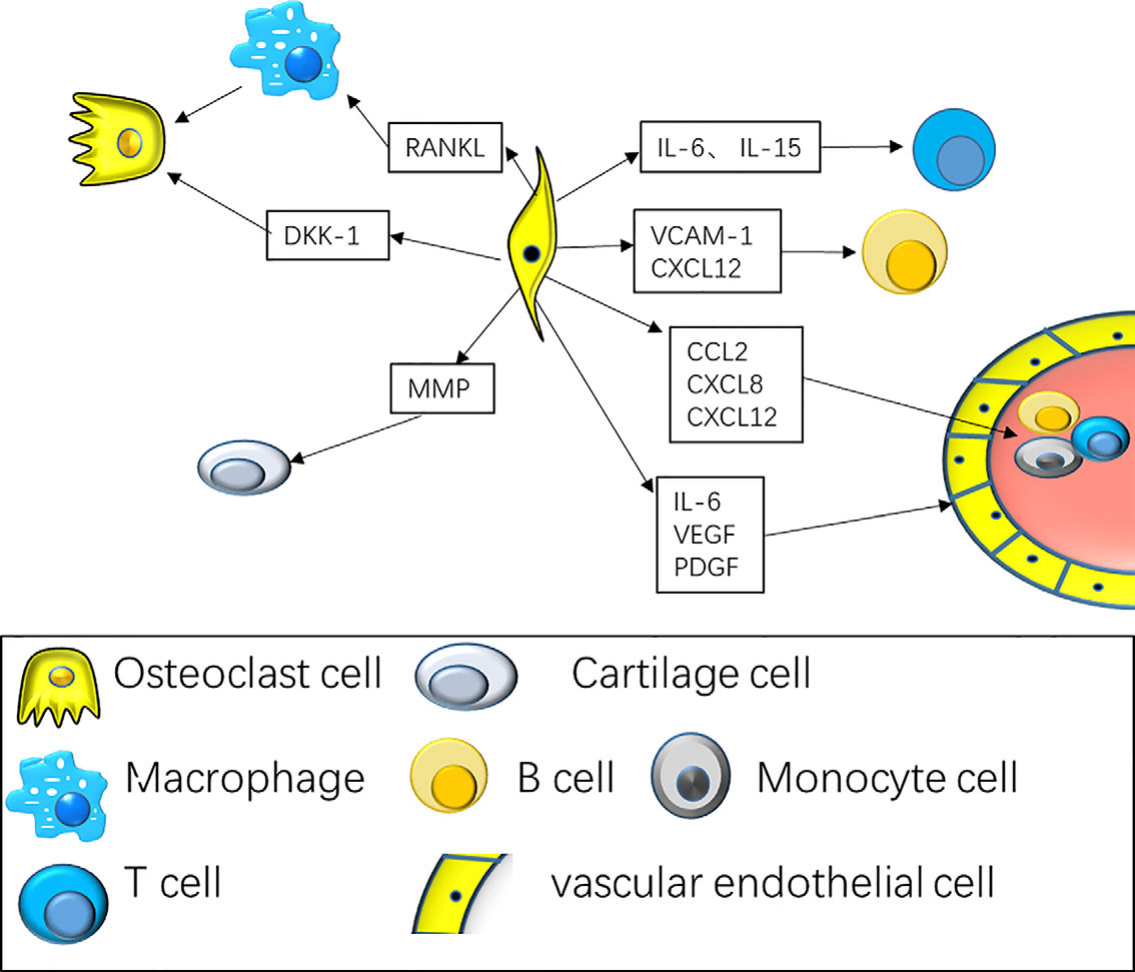
Synovial fibroblasts interact with various types of immune cells to maintain synovitis and continued bone destruction. The interaction of fibroblasts with T cells along with B cells includes the secretion of cytokines consisting of CXCL8, CCL2, CCL5, to promote the mobilization and retention of T cells and B cells. At the same time, fibroblasts cytokines consisting of IL-6 and IL-15 are specifically remarkable for the differentiation of Th17 cell subsets; Fibroblasts promote angiogenesis by secreting VEGF, PDGF. Fibroblasts secrete RANK Ligand, that enhances osteoclast differentiation along with activation resulting in bone erosion, and DKK-1 that represses anabolic osteoblast function, averting repair of bone erosions.

The synovial lining layer goes through remarkable hyperplasia, occasionally reaching a depth of 10–15 cells in RA. The compartment of synovial fibroblast expands in extensive numbers, resulting in remodeling of tissues with formation of papilla. At the same time, synovial fibroblast experiences epigenetic alterations, assuming a stable and activated phenotype, with the ability of infiltrating damaged articular cartilage, as well as the bone (20, 21). The sublining layer likewise expands, with inflammatory cell infiltrates, consisting of macrophages, plasma cells, T cells along with B cells (22). Synovial fibroblasts and macrophages play an indispensable role in joint destruction and disease persistence (23–25). Synovial fibroblasts contribute to disease progression by producing disease-linked cytokines, chemokines, as well as extracellular matrix remodeling components (26, 27) (**Figure 2**).

In RA, synovial fibroblasts can promote inflammation and cartilage destruction, while the interaction between T cells and synovial fibroblasts promotes T cell recruitment and synovial fibroblasts activation (28). The inflammatory cells produced TNF-a to induces vascular endothelial cells and synovial cells to produce CXCL13, which recruits circulating B cells and T cells to the inflammatory site and forms ectopic germinal center. When joint derived helper T cell 17 (Th17) is activated and recruited into the joint to induce inflammatory response, synovial fibroblasts can secrete CCL20 in response to IL-17 and other pro-inflammatory cytokines, and accelerate Th17 cell recruitment to induce and enhance arthritis (29). Studies have shown that there were specific staining of CD4, CXCR5 and ICOS in RA synovial tissue, suggesting that follicular helper T cell (Tfh) cells may exist in synovial tissue. Studies on co-culture of synovial fibroblasts and Tfh cells show that RA synovial fibroblasts can promote the proliferation of peripheral blood Tfh cells by secreting IL-6 (28). IL-21 is mainly secreted by Th17, Tfh and natural killer T cell (NKT). The proliferation of RA-FLS was up-regulated in the presence of IL-21. Tfh cells may promote the proliferation of synovial fibroblasts by secreting IL-21.

Fibroblasts recruit and retain B cells by secreting chemokines (such as CXCL12, CXCL13, VCAM1) and interacting with cell surface adhesion (30). Meanwhile, they secrete BAFF and April to maintain the survival and differentiation of B cells (31). The signal between FLS and B cells is bidirectional. RA-FLS also contributes to the differentiation and activation of B cells, and then B cells can produce a variety of autoantibodies. At the same time, B cells can stimulate RA-FLS to produce more IL-6. Recently, it was show that, the expression of RASF proliferation inducing ligand and B cell activating factor mediated by TLR mediate the survival and function of B cells in RA synovium (32).

It is generally believed that the activation of RA-FLS stimulates peripheral blood monocytes to enter the articular cavity and differentiate into macrophages through paracrine effect (33). In rheumatoid arthritis synovial fluid, macrophages form the largest population of immune cells in rheumatoid arthritis and play a role in synovitis by producing cytokines such as TNF, IL-1 and IL-6 and chemokines such as CCL2 and CXCL8 (34). Alivernini et al (35). shows MerTK^pos^CD206^pos^ STMs cluster can induce the inflammatory response of synovial fibroblasts and promote synovial inflammation by producing pro-inflammatory cytokines and alarm proteins. MerTK^neg^CD206^neg^ STM cluster can produce lipid mediators, induce FLS repair response and alleviate synovial inflammation. FLS can promote the production of RANKL by macrophages and promote osteoclast. The invasion of rheumatoid arthritis synovial fibroblasts is related to the stimulation of IL-1β and the inflammatory response of macrophages. Hypoxia makes RA-FLS secrete more TGF-β and promote macrophages to polarize to M2 and anti-inflammatory factor to play a down-regulation role (36).

### Identified New Synovial Fibroblasts Subsets and Surface Markers

Synovial fibroblasts are the core target cells of a remarkable immune effect in synovial tissue. They interact with lymphocytes, macrophages, as well as other immune cells in the synovium by generating pro-inflammatory cytokines, chemokines along with tissue-destructive factors consisting of IL-6, IL-8 and MMPs (27, 37–39) and play an imperative role in the continuous inflammation and bone destruction of RA synovium (40, 41). Traditional research methods have shown that synovial fibroblasts have certain heterogeneity. Synovial fibroblasts isolated from diverse joints and the same joint lining layer and sublining layer show distinct phenotypes and they are different in their gene expression trends, epigenetic marks and function (42–44). Synovial fibroblasts with different phenotypes have distinct characteristics of adhesion, proliferation, chemotaxis and matrix degradation, as well as different responses to TNF, thus forming a unique microenvironment in each joint (44, 45). At present, a variety of fibroblast surface markers are known, the lining layer fibroblasts are identified *via* the expression of a cell surface biomarker termed as cadherin-11, which allows homotypic adhesion of the lining layer fibroblasts to one another to facilitate the generation of a functional lining layer in the absence of a basal lamina (46, 47). Other biomarkers have also been linked to the lining layer fibroblasts, for instance adhesion molecule VCAM-1, CD55, FAP (fibroblast activation protein), and podoplanin (GP38) (48–50). Sublining fibroblasts are identified with alternative biomarkers, for instance CD90 (THY1) or CD248 (endosialin) and seem to have diverse roles to lining layer fibroblasts (49, 51, 52). With the continuous development of experimental technology, new subsets of synovial fibroblasts are being discovered, and the functions of different subsets are also constantly defined.

Recently, the independent subsets of RA synovial fibroblasts were identified by scRNA-seq technology. There are significant differences in the anatomical position, transcriptome differences and functions of these subpopulations in synovial tissue.

Stephenson et al (53). sequenced 20387 single cells from synovial tissue of 5 patients with rheumatoid arthritis, and found three different subpopulations of RA synovial fibroblasts, which can be divided into two groups according to different surface markers, one is CD55+ synovial fibroblast, the other is CD90+ subset. CD55 is a glycosylphosphatidylinositol-anchored complement-modulating protein (decay-accelerating factor), expressed by synovial fibroblasts with high local abundance in the intimal lining layer, which can protect synovium from immune complex mediated arthritis (48). CD55+ fibroblasts locate to the intimal lining and are responsible for synovial fluid formation and turnover. Of note, hyaluronan synthase 1 (HAS1) (53, 54), lubricant PRG4 (55) and DNASE1L3 were highly expressed. Previous studies have shown that lubricin/proteomeglycan-4 (PRG4) is a mucus glycoprotein secreted by synovial fibroblasts and superficial chondrocytes, which has a variety of homeostasis effects in the joint and play an anti-inflammatory role by combining with TLR2 and TLR4 (56). HAS is mainly divided into three subtypes: HAS1, HAS2 and HAS3, and the three subtypes are independent and differentially regulated, play a different role in arthritis. Among them, TGF-β upregulates HAS1 mRNA (57, 58). Furthermore, Go enrichment analysis indicated that CD55+ fibroblasts expressed functional modules linked to endothelial cell proliferation and modulation of reactive oxygen species responses (53). CD90+ fibroblasts are mainly located in the lower layer of synovial sublining layer, which are enriched for modules linked to metallopeptidase activity, as well as the organization of the extracellular matrix (53).

**Figure 3:**
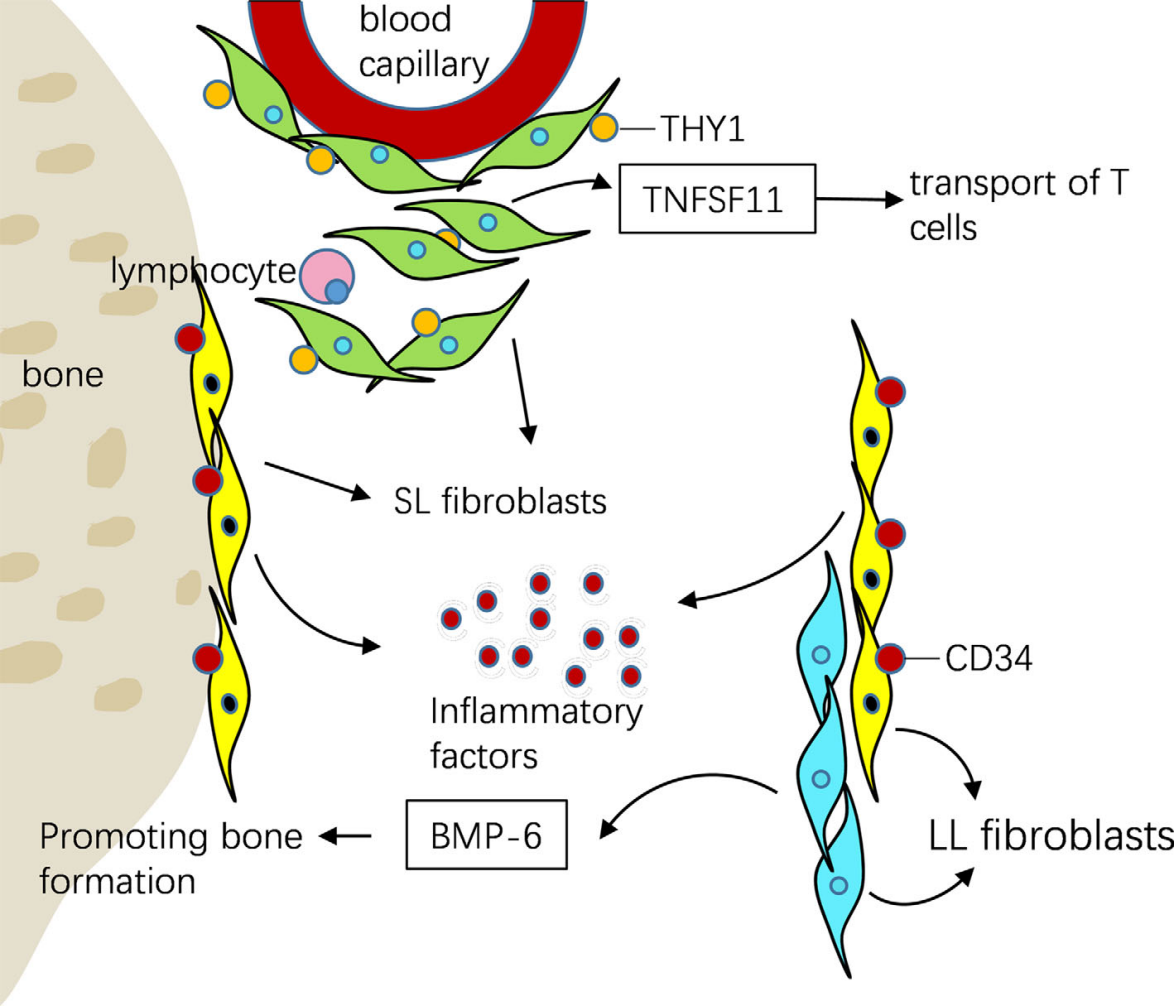
ScRNA-seq showed that three different fibroblasts were distributed in different parts of synovial tissue and play different roles in RA. CD34+ fibroblasts were reported in superficial lining, as well as deeper sublining areas of the synovium. They promoted the progress of joint inflammation by secreting a large number of inflammatory factors. CD34–THY1– fibroblasts were remarkably reported in lining area and express BMP-6, recognized to enhance osteoblastic bone formation. CD34–THY1+ fibroblasts in RA produce a discrete perivascular zone that surrounds capillary structures in the deep sublining layer of the synovium, it participate in the transport of T cells by overexpression of TNFSF11.

Mizoguchi et al. (59) identify seven different fibroblast surface protein phenotypes and classified them into three subsets according to the expression of podoplanin (PDPN) (60, 61), cadherin-11 (CDH11) (46, 62), THY1 (also known as CD90) (63) and CD34 (64) by integrating transcriptomic data. they found that different subsets of fibroblasts play different roles in joint inflammation and bone destruction. CD34, a remarkable marker of stem/progenitor cells, is an intercellular adhesion molecule. It is expressed in some synovial fibroblasts (65), and also in endothelial cells and nerve tissues (66). CD34+ fibroblasts were reported in superficial lining, as well as deeper sublining synovium areas. They proliferated actively in the synovium of RA. At the same time, they promoted the progress of joint inflammation by secreting a large number of inflammatory factors and mediating the enrichment of leukocytes, which was related to the migration of fibroblasts (59). CD34–THY1– fibroblasts were remarkably reported in lining area and express BMP-6, thought to enhance formation of osteoblastic bone (59, 67). in RA patients the count of CD34–THY1+ fibroblasts is threefold than that in OA, they generate a discrete perivascular zone, which surrounds the capillary structures in the synovium deep sublining layer, especially near aggregations of lymphocytes, and play an indispensable role in matrix infiltration, immune cell mobilization and osteoclast formation. At the same time, through its overexpression of TNFSF11 [also referred to as receptor activator of nuclear factor-κB ligand (RANKL)] (68, 69), CD34–THY1+ fibroblasts participate in the transport of T cells in autoimmune inflammation. Therefore, this subgroup may also be responsible for RA lymphocyte accumulation in synovium (59). Transcriptome sequencing and scRNA-seq have the same conclusion, which indicates that gene differences reflect biological differences rather than technical or random differences (59) (**Figure 3**).

In the larger study, the synovial tissues of 51 RA or OA patients were used for single-cell sequencing and transcriptomics to analyze the cell subsets associated with arthritis (55). Among 1844 synovial fibroblasts, four presumed subpopulations were identified: CD34+ fibroblasts, HLA-DRAhi fibroblasts, and Dkk3+ fibroblasts were located in the sublining layer, while CD55+ fibroblasts were located in the lining layer (55). HLA-DRAhi fibroblasts highly express CXCL12 and HLA-DRA, and is the main source of IL-6. IL-6 is mainly secreted from pro-inflammatory M1 macrophages along with RA synovial tissue fibroblasts (70, 71). Along with IL-1β, IL-6 has an imperative role for Th17 cell differentiation, which is remarkable in RA angiogenesis (72–75). At the same time, IL-6 can activate Tfh by up regulating signal transducer and activator of transcription (STAT-1 or STAT-3), depending on the expression of bcl-6 (76). The antagonists of TNF-a and IL-1B can down regulate the number of Tfh cells by reducing the level of IL-6 (28). The RA fibroblasts stimulated by adiponectin can promote the production of TFH cells by producing IL-6. Intra articular injection of ad aggravates synovial inflammation and increases the frequency of Tfh cells in CIA mice (77). HLA-DR is a class of MHC-II, which is considered to present exogenous antigen. HLA-DR and CD68 are co-expressed in Macrophage-like synoviocytes (78). DKK3+ fibroblasts was a novel subtype of sublining fibroblast, characterized by elevated expression of DKK3, CADM1, and COL8A2, and can prevent cartilage degradation *in vitro* (55, 79). These subsets may be the key inflammatory subsets of RA.

### To reveal the New Function of Different Subsets of Synovial Fibroblasts

Chronically activated fibroblasts account for the degradation of excessive matrix, destroying cartilage, resulting in permanent joint damage in RA by activates osteoclasts (80). Targeted treatment of synovial fibroblasts may become an effective strategy for the treatment of RA. Recent studies have shown that Notch signaling plays a role in THY1 expressing perivascular and subcutaneous fibroblasts, and blocking Notch3 signaling can alleviate joint inflammation in mice (81). Another study showed, specific small molecule agonist can activate melanocortin type 1 receptor (MC11), make synovial fibroblasts activate GPCR and aging, so as to promote the regression of arthritis (82).

Mizoguchi et al. (59) found that different fibroblast subpopulations play different roles in joint inflammation and bone destruction.CD34–THY1+ fibroblasts are abundant around blood vessels in RA synovium, with their expression profile revealing prospective pathogenic functions in matrix infiltration, immune cell mobilization, and osteoclastogenesis. CD34–THY1+ fibroblasts are located around the vessels under the synovial lining, which are related to the activity of disease, the infiltration of immune cells and the increase of bone destruction. In RA, a remarkable pathogenic effector role of synovial fibroblasts is modulation of osteoclastogenesis, a process dominantly carried out by TNFSF11 and opposed by TNFRSF11B [also referred to as osteoprotegerin (OPG)], which is a decoy receptor for TNFSF11. An exploration of the genes linked to osteoclastogenesis exhibited high expression of TNFSF11, but low TNFRSF11B expression in CD34–THY1+ fibroblasts.

FAPα, a cell-membrane dipeptidyl peptidase (83), was remarkably higher in synovial tissue along with cultured synovial fibroblasts isolated from individuals who fulfilled classification criteria for RA in contrast with patients in whom joint inflammation resolved, implying that FAPα expression might be linked to a pathogenic fibroblast phenotype (84, 85). A new study in 2019 (2) showed that FAP α was expressed in the sub-lining and lining layer fibroblasts. According to the expression of THY1, synovium can be divided into FAPα+THY1+ lining layer fibroblast and FAPα+THY1- sublining fibroblast. Five subtypes of fibroblasts were identified by single cell sequencing of inflammatory joints in RA mice, and similar results were obtained in synovium of RA patients. Further research shows that, FAPα+THY1+ fibroblasts mediate synovial inflammation by secreting cytokines and chemokines, while FAPα+THY1− plays a role in bone destruction by expressing osteoclast activity inducers consisting of matrix metalloproteinases, suggesting that synovial fibroblasts at different anatomical positions play different roles in the pathogenesis of RA.

### Dynamic Changes of Synovial Fibroblast Subsets in Inflammatory State

The degree of synovial cells and immune cells infiltrate is a highly variable phenomenon in different disease stages and drug exposure in RA (86–88), the degree of synovitis is related to the clinical phenotype of RA and as such has been explored as a prospective source of predictive, as well as prognostic biomarkers in RA (88). Recent research shows that different subsets of synovial fibroblasts play an independent role in the process of disease. Therefore, scholars speculate that the proportion of fibroblast subpopulations related to disease may be different in different diseases and different stages of RA. ScRNA-seq also showed the dynamic changes of synovial fibroblast subsets in inflammatory state.

Mizoguchi et al. (59) showed that there was a significant difference in the proportion of the three synovial fibroblast subsets between OA and RA synovium, CD34–THY1+ fibroblasts accounted for 22% of the total fibroblasts in RA and 8% in OA. There were less CD34-THY1-, more CD34-THY1+ and more CD34+ fibroblasts in swollen joints. In addition, the proportion of CD34-THY1+ fibroblasts was correlated with the proportion of leukocyte infiltration, synovitis and synovial hypertrophy, which indicated that the altered proportion of fibroblast subsets in RA reflected the molecular level and clinical level of tissue inflammation. Huang et al. (89) showed that some common fibroblast markers, consisting of procollagen I (COL1A1), Prolyl-4-hydroxylase (P-4-H), Vimentin, along with procollagen III (COL3A1), are different in OA FLS and RA FLS. Besides, RA FLS exhibits more severe cellular behavior in contrast with OA FLS, entailing a more rapid rate of proliferation, stronger invasive potential, and elevated expression as well as secretion of inflammatory cytokines. Additionally, elevated expression of inflammatory markers, consisting of CCL2, IL-6, IL-1β and TNF-α, were also reported in RA FLS in contrast with FLS isolated from the less inflamed OA synovium. This suggests that different subsets of fibroblasts may play a role in the pathogenesis of OA and RA.

The continuous progress of ScRNA-seq technology has opened the era of exploring new targeted drugs for synovial fibroblasts. At present, there are no specific drugs targeting synovial fibroblasts. Therefore, identifying the heterogeneity of fibroblasts and pathogenic fibroblast subsets to determine the disease-related fibroblast subsets that can be used as specific targets for disease treatment may provide new effective strategies for treating RA (90, 91). Single cell sequencing of synovial fibroblasts (55) will also serve as a research template to identify pathogenic interstitial fibroblast subsets in other autoimmune diseases, for instance connective tissue disease related pulmonary interstitial disease. 

## Single-Cell RNA-Sequencing of Synovial Macrophage

Previous studies have shown that the aggregation of monocyte macrophages can promote the occurrence and development of arthritis, and the infiltration of synovial macrophages is positively correlated with the progress of joint destruction (25, 92). With the development of research technology, the research on the heterogeneity of synovial macrophages is also in-depth. ScRNA-seq leads to different understanding of synovial macrophages, which have heterogeneous subpopulations with different sources and biological characteristics.

### Origin and Biological Heterogeneity of Synovial Macrophage Subsets

For numerous years, it was speculated that macrophages primarily originated from circulating monocytes differentiation, however recently morphological along with functional differences between these cells dispute this speculation (93, 94). Nonetheless, a series of recent reports have documented that the origins of macrophages in different tissues/organs are not exclusively originated from circulating monocytes. Tissue-resident macrophages derive mainly from embryonic progenitors and to less degree from intermediates of circulating monocytes, additionally many of them are capable of self-renewal (95–98). The mobilized population of monocyte originated macrophages significantly increase during inflammatory conditions. It is generally believed that RA-FLS can stimulate peripheral blood monocytes to enter articular cavity and differentiate into macrophages through paracrine effect after activation (33). By the different transcriptional and epigenetic characteristics showed in macrophages different subsets, the unique functional patterns of variant tissue were indicated. It was illustrated that embryonic macrophages participate in tissue remodeling, while adult-originated macrophages mainly assist in host defense.

Macrophages constitute remarkable plastic cells, with the ability switch from one phenotype to another (99). Synovial macrophages can be divided into different specific subsets according to their origins and functions. Two primary macrophage subpopulations with different roles consist of classically activated or inflammatory (M1) and alternatively activated or anti- inflammatory (M2) macrophages have been recognized (100). M1 macrophages that mediate resistance to pathogens and tissue destruction by produce pro- inflammatory cytokines like TNF, IL-6 and IL-1β,CCL2, IL-8, IL-12 and IL-23 (101, 102); M2 macrophages can remove debris and promote tissue repair by produce anti-inflammatory cytokines consisting of TGF-β, IL-10, IL-4, IL-13 (103). In RA, there is a positive correlation between the infiltration of synovial macrophages and the progress of joint destruction (92). The imbalance of M1/M2 in synovium is one of the remarkable reasons for chronic synovitis. Misharin et al. (104) showed that macrophages can be transformed from M1 to M2 in the progression of RA to promote damage repair. After co-culture of RA-FLS and macrophages in normoxia, the cytokines secreted by RA-FLS can strongly inhibit the pro-inflammatory activity of M1 and enhance the expression of genes promoting M2 polarization (105). Deciphering the process of macrophage polarization, mobilization, and functions may provide insights for the development of new therapies for RA. At the same time, Zhang et al. (106) showed that Macrophages with M1 polarization gather in synovium of OA patients and OA model mice. M1 polarization of macrophages promotes synovium hyperplasia, synovitis and progression of OA. Controlling the polarization of synovium macrophages to M2 may be a new strategy for prevention and treatment of OA.

In the latest study (107), Two types of SM were found SM after tracking macrophages in mice: embryonic SMs (ESMs), and bone marrow-derived SMs (BMSMs). ESM expressed anti-inflammatory cytokines, consisting of IL-4 and IL-10, while BMSM expressed pro-inflammatory cytokines, consisting of IL-1β and TNF. In arthritic mice, the number of ESMS reduced during disease development and then increased during regression, whereas BMSM was the opposite. This study first confirmed that synovial macrophages have at least two origins, ESM and BMSM, and their effects are different. Secondly, this study also showed that two types of SM also exist in the synovium of RA patients and have similar anti-inflammatory and anti-inflammatory phenotypes of ESM and ESM. Through the different cell characteristics and dynamic expression patterns in RA patients/CIA mice, it was revealed that two subpopulations of different origins, embryonic ESM (anti-inflammatory) and bone marrow derived BMSM (pro-inflammatory), play different roles in arthritis.

In Monocyte of human RA synovium, IL1B+ pro-inflammatory monocytes (SC-M1), NUPR1+ monocytes (SC-M2), C1QA+ monocytes (SC-M3), and interferon (IFN) activated monocytes (SC-M4) were identified by Single cell sequencing. Among them, SC-M3 and SC-M4 matched with those of mouse resident synovial macrophages, while SC-M1 and SC-M4 were similar to those of mouse monocyte derived synovial macrophages (55).

To elucidate the immunomodulatory mechanism of drug-free remission in RA, Alivernini et al. (35) analyzed 32000 STMs of patients with early/active RA, refractory/active RA, and RA in persistent remission using single-cell transcriptional analysis to identify the phenotypic changes. MerTK^pos^CD206^pos^ and MerTK^neg^CD206^neg^ STM contain nine different clusters, which can be divided into four subgroups with different functions of homeostasis, regulation and inflammation. Compared with the healthy control group, the number of MerTK^pos^CD206^pos^ increased in remission stage and MerTK^pos^CD206^pos^ cluster increased in active stage. Therefore, the tissue residence of MerTK^pos^ CD206^pos^ STM seems to play an important role in maintaining the sustained remission of inflammation. The decrease of MerTK^pos^ STM proportion in remission stage is related to the increased risk of disease after drug withdrawal. Therefore, the regulation of mertkpos STM may be a potential treatment for RA. Other studies have shown that the subsets of HBEGF+ inflammatory macrophages were enriched in RA tissue and formed by resident fibroblasts and cytokine tumor necrosis factor (TNF). Other studies have shown that the subsets of HBEGF+ inflammatory macrophages are enriched in RA tissue and contribute to fibroblast mediated joint destruction (108).

### Synovial Macrophages Created a Protective Barrier for the Joint

ScRNA-seq can map a single cell of a given lineage to a unified orbit, so as to clarify the time sequence of cell development and differentiation and infer the development trajectory of cells from a new perspective by using this cross-time continuum. Known as “pseudotime” (109).

In 2019, Gerhard et al. (110) first discovered a new subpopulation of synovial macrophages, which serves as a protective and tightly connected barrier of synovial macrophages. The researchers used a variety of methods, including single cell sequencing, to study the subsets and functions of macrophages in different states. CX3CR1 is a kind of chemokine receptor that can be used by monocytes, CX3CR1+ lining macrophages and CX3CR1- interstitial macrophages are two kinds of tissue-resident macrophages origin from the embryo, among them, CX3CR1- stromal synovial macrophages can be divided into specific subsets according to the expression of AQP1, MHCII and RELM-α. Both RELM α+ macrophages and CX3CR1+ lining macrophages are derived from proliferative MHCII+ interstitial macrophages (110). This study found that CX3CR1+ tissue resident macrophages derived from CX3CR1 monocytes form an internal immune barrier in synovial lining and physically isolate joints (110).

Using an arthritis mouse model in which macrophages might be tracked *via* engineering them to be fluorescent, the authors reported that the barrier layer was remarkably dynamic. Upon inducing arthritis, the layer experienced active remodeling causing loosening of the physical cross talks between lining-layer fibroblasts and barrier macrophages. The barrier macrophages might ingest and remove inflammatory immune cells called neutrophils that aggregate and die in the synovial fluid in arthritis. These CX3CR1+ macrophages in the lining layer of synovium provide tight junction mediated shielding for intra-articular structures, forming a protective macrophage barrier and restricting inflammatory response.

When comparing the single-cell RNA data from mice with similar data sets available from an assessment of the joints of individuals with RA, the gene-expression trends of the macrophage subsets matched up between the two species. This implies that cells, which are similar to the barrier, as well as interstitial macrophages in mice could similarly exist in humans, and hence be relevant to human disease. This study reveals the unexpected functional diversity among the synovial macrophages, and the new subpopulation of the protective barrier effect of the synovial macrophages, which is of new significance for the study of the heterogeneity of macrophages in health and disease.

## Reviews and Perspectives

The continuous application of ScRNA-seq technology can help us to identify the heterogeneity of cells so as to determine the targets related to the treatment of RA, and also play an important role in tracking the lineage or development relationship between synovial cells and immune cells, osteoclasts and other cells, so as to further discover the synovial fibroblasts, synovial macrophages and other immune cells in RA and making the study of new subpopulations, differentiation and dynamic evolution of cells more in-depth. The combination of scRNA-seq technology and bioinformatics technology can effectively interpret the gene expression, splicing and other information obtained by sequencing, and construct the regulatory network based on these information, so as to analyze the role of synovial tissue in the occurrence and development of RA. ScRNA-seq technology still has great application space in the research of autoimmune diseases such as RA. In particular, the development of high-throughput single-cell RNA sequencing platform has carried out cell-to-cell difference analysis and exploration of potential mechanisms from multiple dimensions, and can predict the activity and radiological progress of RA from the characteristics of cell and molecular mechanism of synovial tissue, and predict the clinical efficacy of csDMARD treatment (111), which will bring greater breakthrough for RA treatment.

## Author Contributions

LC drafted the manuscript, drew illustrations, and discussed the content with the other authors. CW conceived the topic of the manuscript and revised the content of the manuscript. YW, RW, TD, and HX revised the manuscript. CG and XL also critically revised the content of the manuscript. All authors contributed to the article and approved the submitted version.

## Funding

This work was supported by the National Natural Science Foundation of China (No. 81971543), National Natural Science Foundation of China (No. 8197061587), National Natural Science Foundation of China (No. 81471618), and Key Research and Development (R&D) Projects of Shanxi Province (201803D31119).

## Conflict of Interest

The authors declare that the research was conducted in the absence of any commercial or financial relationships that could be construed as a potential conflict of interest.
